# Seroprevalence of Norovirus Genogroup IV Antibodies among Humans, Italy, 2010–2011

**DOI:** 10.3201/eid2011.131601

**Published:** 2014-11

**Authors:** Barbara Di Martino, Federica Di Profio, Chiara Ceci, Elisabetta Di Felice, Kim Y. Green, Karin Bok, Simona De Grazia, Giovanni M. Giammanco, Ivano Massirio, Eleonora Lorusso, Canio Buonavoglia, Fulvio Marsilio, Vito Martella

**Affiliations:** Università degli Studi di Teramo, Teramo, Italy (B. Di Martino, F. Di Profio, C. Ceci, E. Di Felice, F. Marsilio);; National Institutes of Health, Bethesda, Maryland, U.S.A. (K.Y. Green, K. Bok);; Università degli Studi di Palermo, Palermo, Italy (S. De Grazia, G.M. Giammanco);; Azienda USL di Reggio Emilia, Reggio Emilia, Italy (I. Massirio);; Università Aldo Moro di Bari, Valenzano, Italy (E. Lorusso, C. Buonavoglia, V. Martella)

**Keywords:** noroviruses, NoVs, genogroups, GIV NoVs, carnivores, zoonoses, antibodies, humans, interspecies transmission

## Abstract

Noroviruses (NoVs) of genogroup IV (GIV) (Alphatron-like) cause infections in humans and in carnivorous animals such as dogs and cats. We screened an age-stratified collection of serum samples from 535 humans in Italy, using virus-like particles of genotypes GIV.1, circulating in humans, and GIV.2, identified in animals, in ELISA, in order to investigate the prevalence of GIV NoV-specific IgG antibodies. Antibodies specific for both genotypes were detected, ranging from a prevalence of 6.6% to 44.8% for GIV.1 and from 6.8% to 15.1% for GIV.2 among different age groups. These data are consistent with a higher prevalence of GIV.1 strains in the human population. Analysis of antibodies against GIV.2 suggests zoonotic transmission of animal NoVs, likely attributable to interaction between humans and domestic pets. This finding, and recent documentation of human transmission of NoVs to dogs, indicate the possibility of an evolutionary relationship between human and animal NoVs.

## Abstract 2

Antibodies specific to genogroup IV identified in human specimens suggest zoonotic exposure.

Noroviruses (NoVs) are a major cause of epidemic gastroenteritis in children and adults. They cause nearly half of all gastroenteritis cases and >90% of nonbacterial gastroenteritis epidemics worldwide (*1*). NoVs belong to the genus *Norovirus* in the family *Caliciviridae* (*2*,*3*). NoV virions are nonenveloped and ≈30 to 35 nm in diameter. The icosahedral capsid surrounds a 7.7-kb positive-sense single-stranded RNA genome covalently linked to viral protein g (VPg) at the 5′ end and polyadenylated at the 3′ end (*4*). The RNA genome is organized into 3 open reading frames (ORFs). ORF1 encodes a polyprotein that is cleaved by the virus-encoded protease to produce several nonstructural proteins, including the RNA-dependent RNA polymerase; ORF2 encodes a major capsid protein, VP1; and ORF3 encodes a small basic protein (VP2) that has been associated with the stability of the capsid (*4*,*5*). Based on the full-length VP1 aa sequence, NoVs have been divided into 6 genogroups (GI to GVI) and multiple genotypes (*6*,*7*). However, only GI, GII, and GIV NoVs have been shown to infect humans; GII strains are the most prevalent worldwide (*4*). Human GIV NoV (Alphatron-like) strains have been identified at low prevalence from either sporadic cases or outbreaks of human gastroenteritis (*8*–*10*). However, analysis of wastewater, sewage, and seafood in Japan and Italy has revealed, indirectly, that GIV NoVs are common in humans (*11*–*14*).

GIV.2 NoVs (strain GIV.2/Pistoia/387/06/ITA) were first detected in the feces of a captive lion cub with severe hemorrhagic enteritis in Italy (*15*). Subsequently, similar NoVs were identified in fecal samples of dogs and cats with diarrhea (*16*,*17*). Sequence comparison in the VP1 of human and animal GIV NoVs has revealed that, although they are genetically related, the 2 groups of viruses represent 2 distinct genotypes: GIV.1 viruses predominate in humans and GIV.2 in animals (*7*,*15*). Historical evidence shows that viruses genetically and antigenically closely related to human NoVs might infect animals (*15*–*19*). Also, a human GII.4 NoV strain has been found to replicate and cause clinical signs and lesions in experimentally-infected gnotobiotic pigs and calves (*20*,*21*). These findings have raised public health concerns about potential cross-species transmission and generation of novel human NoV strains by recombination. The close genetic relatedness (*17*,*22*) of human and animal GIV NoVs indicates that they may have originated from a common ancestor. Interspecies transmission between humans and pets might have been facilitated by the social interactions established since domestication of small carnivores. This eventuality has been demonstrated firmly in a recent study in Finland, which reported the detection of GII.4 and GII.12 NoVs in the dogs belonging to human patients hospitalized with NoV gastroenteritis (*19*). To address whether cross-species transmission of GIV.2 might occur between carnivores and humans, we investigated the prevalence of antibodies against GIV.1 and GIV.2 NoVs in a representative population in Italy spanning all age groups.

## Materials and Methods

### Human Serum Samples

Human serum samples were collected from a random sampling of inpatients and outpatients seeking medical attention for various clinical conditions at the Microbiology Unit of the University Hospital “P. Giaccone” of Palermo, Sicily, Italy, during September 2010–June 2011. All patients were enrolled in the study after giving informed consent. Serum samples from a total of 535 persons were tested. For our analysis, samples were divided on the basis of patient age groups: <1 year, 1–5 years, 5-year age groups from 6–79, and >80 years of age.

### Virus-like Particles

The recombinant baculoviruses carrying the genes for the viral capsid proteins of the lion/NoV/GIV.2/Pistoia/387/06/ITA and Hu/NoV/GIV.1/SaintCloud624/1998/U.S. strains were obtained as previously described (*23*,*24*). For large-scale production of virus-like particles (VLPs), 100 mL of *Sf9* cells (1 × 10^6^ cell/mL) suspension culture were inoculated with the recombinant baculovirus at a multiplicity of infection of 3 PFU/cell. Assembled VLPs were isolated from the culture medium of infected cells at 48 h postinfection by centrifugation at 4,000 rpm for 20 min. The recombinant capsid proteins were concentrated by ultracentrifugation through a 17% sucrose cushion in 50 mmol/L Tris-HCl, pH 7.5; 1 mmol/L EDTA; and 100 mmol/L NaCl, aka TEN-buffer, and purified on a discontinuous 20%–60% (wt/vol) sucrose gradient, as previously described (*24*). The collected fractions were dialyzed against phosphate-buffered saline (PBS), and the protein concentration of VLP preparations was determined by measuring the optical density at 280 nm (OD_280_) and visually by running aliquots containing bovine serum albumin standards on sodium dodecyl sulfate–10% polyacrylamide gel electrophoresis. The presence of VLPs was confirmed by electron microscopy.

### Antigenic Relationships of VLPs

To evaluate the antigenic relationship between GIV.1 and GIV.2 VLPs, we tested polyclonal rabbit serum produced against the lion GIV.2 strain (*24*) for GIV.1 and GIV.2 antigen reactivity by using Western blot (WB) testing to limit dilution analysis (not shown). Although a modest reactivity with the heterologous GIV.1 antigen was observed at dilution ≤1:100, the GIV.2 antiserum showed the highest levels of reactivity with the homologous antigen. Two experiments were performed to investigate serologic cross-reactions between GIV VLPs and human NoVs belonging to genetic groups GI and GII. First, we tested GIV.1 and GIV.2 VLPs using an antigen-ELISA kit (Ideia Norovirus, Oxoid, Basingstroke, UK). Second, we assessed the reactivity of the GIV.2 antiserum with GII.4 VLPs (Hu/NoV/GII.4/MD145–12/1987/U.S.) (*25*) in WB analysis. The GIV.1 and GIV.2 VLPs were not detected by the commercial antigen-ELISA kit even at concentrations <10 μg of protein/mL; the GIV.2-specific rabbit antiserum did not show reactivity with GII.4 VLPs in WB analysis.

### ELISA

For the development of the antibody detection ELISA, we diluted the supernatant containing mock infected cells GIV.1 and GIV.2 VLPs to a final concentration of 1 μg/mL in carbonate-bicarbonate buffer (0.05 M, pH 9.6) and 100 μL was added to each well of a 96-well EIA plate (Costar, Bio-Rad Laboratories, Segrate, Italy). The plates were incubated at 4°C overnight. The wells were washed 5 times with 0.1% Tween-PBS (PBS-T) and then blocked with 200 μL of PBS containing 2% bovine serum albumin at room temperature for 2 hours. After the 5 washings, each serum sample (100 μL), diluted to 1:100 in 1% dried milk (Blotto, Santa Cruz Biotechnology, Inc., Heidelberg, Germany) in PBS, was added to the antigen-coated wells, and the plates were incubated at 37°C for 1 h. Plates were washed 5 times with 0.1% PBS-T and then incubated with horseradish peroxidase-conjugated goat anti-human IgG (Sigma-Aldrich, Milan, Italy) at 1:5,000 dilution for 30 min at 37°C. The reaction developed after the addition of 100 µL per well of 2,2′-azino-di-(3-ethylbenzthiazoline-6-sulfonate) substrate for 15 min and stopped after addition of an equal volume of 1 M/L phosphoric acid. We measured absorbance at 405 nm using a Multiskan automatic plate reader (ThermoLabsystems, Abu Gosh, Israel). The cutoff point of the ELISA was established as the mean of the OD_405_ readings of 50 human serum samples negative in WB for both GIV.1 and GIV.2 antigens plus 2 standard deviations. For each tested sample, a positive/negative ratio (OD_405_ of VLPs/OD_405_ of mock infected cells) ≥2.0 was used to evaluate the background binding. All samples that had OD_405_ values ≥0.5 at the initial dilution of 1:100 were considered positive and titrated in 2-fold dilutions. Mean ELISA antibody titers were calculated and expressed as the reciprocal of the highest serum dilution that had positive absorbance (OD_405_≥0.5) for GIV.1 and/or GIV.2 antigens. The data were analyzed by using GraphPad Prism Software (GraphPad Software, La Jolla, CA, USA). We used a χ^2^ test for trend to determine the trend of age-class prevalence of IgG antibodies to GIV.1 and GIV.2 VLPs, and Fisher exact test to determine the difference between the seroprevalence rates for the 2 antigens and the differences in prevalence among the age groups. A p value of <0.05 was considered statistically significant.

## Results

Of 535 human serum samples tested at the initial dilution of 1:100, 151 (28.2%) were positive for the presence of GIV NoV-specific antibodies: 107 (20.0%) samples reacted with both GIV.1 and GIV.2 VLPs, 39 (7.3%) with GIV.1, and 5 (0.9%) with GIV.2. When all the positive serum samples were rescreened by endpoint titration, 118 samples (22.0%) reacted with the GIV.1 antigen at rates of 14.5% (78/535) at dilution of 1:200 and 7.4% (40/535) at dilution of 1:400. Twenty-six (4.8%) samples reacted with GIV.2 at rates of 2.2% (12/535) at a dilution of 1:200 and 2.6% (14/535) at 1:400. Seven (1.3%) samples were positive for antibodies against the antigens tested at final dilutions of 1:200 and 1:400 (3 and 4 samples, respectively) (Table).

**Table:** Seroprevalence of IgG antibody against norovirus GIV.1 and GIV.2 in human serum specimens, Italy, 2010–2011*

NoV GIV virus-like particles	Serum dilutions		Total (%)
	1:200 (%)	1:400 (%)	
GIV.1	78 (14.5)	40 (7.4)	118/535 (22.0)
GIV.2	12 (2.2)	14 (2.6)	26/535 (4.8)
GIV.1+GIV.2	3 (0.5)	4 (0.7)	7/535 (1.3)

*GIV; genogroup IV; NoV, norovirus.

We further examined age-related patterns of seroprevalence for GIV.1 and GIV.2 NoVs (Figure). The rate of IgG antibodies against GIV.1 NoVs was 6.6% in infants <1 year of age. This rate of GIV-reactive serum samples increased from 6.6%–37.9% in the 1- to 5-year age group and reached a peak of 43.3% in children 6–10 years of age. The prevalence then declined, reaching the lowest values in the 11- to15-year age group and in young adults who were 26–30 years of age (10.7% and 9.6%, respectively). The rate of GIV.1-positive NoVs gradually increased from 14.9% in the 31- to 35-year age group to 44.8% in the 61- to 65-year age group. The prevalence in the older age groups gradually declined to 10.0% in persons >80 years of age.

**Figure:**
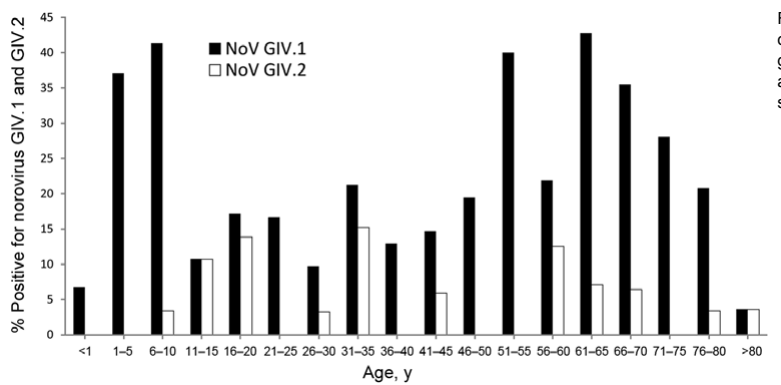
Age-related prevalence of antibodies against norovirus genogroup IV, genotypes GIV.1 and GIV.2, in human serum specimens, Italy, 2010–2011.

Compared with prevalence of IgG antibodies against GIV.1 NoVs, the overall prevalence for IgG antibodies against GIV.2 NoVs was low in most age groups, varying from 6.8% in preschool-age children (1–5 years) to 10.7% in the 11- to 5-year age group, reaching peaks in young adults who were 16–20 years (13.8%) and 31–35 years of age (15.1%), and its prevalence ranged 9.37%–12.5% in persons 56–70 years of age. The lowest level of seropositivity was in persons in the age groups 26–30, 41–45, and 76–80 years (3.2%–5.9%, respectively). Antibodies against GIV.2 NoVs were not identified in samples from the following age groups: <1 year, 21–25 years, 36–40 years, 46–55 years, and 71–75 years.

To perform statistical analysis, we combined data from several age groups because, in some age groups, no serum samples tested positive for GIV.2 NoVs, and we found that the positive GIV.1 trend, when charted, showed a significant shape (χ^2^ = 16.50 p<0.01) of seroprevalence. Two peaks were observed in the age groups 1–10 and 56–70 years. Likewise, statistically significant differences were found for the GIV.1 seroprevalence in age group 1–10 years when compared with that in the age groups 11–25 (p = 0.03) and 26–40 (p = 0.02) years. The differences were not significant when the GIV.1 seroprevalence in age groups 1–10 years was compared to that in the age groups 41–55 (p = 0.38), 56–70 (p = 0.61,), and >70 years (p = 0.06).

Similar analyses were performed for GIV.2 NoV antibodies, but no statistical significance was found (Χ^2^ = 10.74, p = 0.0568), likely because of the low number of seropositive persons. Two peaks in age groups were also observed for GIV.2 NoV antibodies: in the age groups 56–70 and 11–25 years. When comparing the seroprevalence rates between the NoV antigens GIV.1 and GIV.2, statistically significant differences were observed (p<0.01), suggesting that the seroprevalence values reported for the human serum specimens are not related.

## Discussion

In vitro expression of viral proteins is essential to gathering information on the epidemiology of noncultivable NoVs in humans and animals (*26*–*28*). Serologic studies documenting the seroprevalence of GI and GII NoVs in humans have been performed since the early 1990s (*27*,*29*,*30*); however, serologic studies on GIV NoVs have not been performed, thus limiting the understanding of the epidemiology of viruses in this genogroup.

We examined the prevalence of IgG antibodies against GIV.1 and GIV.2 NoVs and found an overall seroprevalence rate of 28.2%. The majority of the positive serum samples (OD_405_ ≥0.5) reacted at the initial dilution of 1:100 with both GIV.1 and GIV.2 VLPs. This could be accounted for by the existence of highly conserved epitopes between genotype GIV.1 and GIV.2 NoVs. Heterologous sero-responses among strains of the same genogroups have been reported frequently in ELISA-based investigations (*31*,*32*), although greater sero-responses were detected against the homologous strains (*33*). Accordingly, to rule out the cross-reactivity between GIV.1 and GIV.2 NoVs, all the samples with OD_405_ values ≥0.5 were further assessed by endpoint titration. Antibodies specific for GIV NoV genotypes GIV.1 and GIV.2 were detected. These findings support previous hypothesis that humans may be exposed to NoVs from carnivores (*34*) or to antigenically related strains.

In general, the seroprevalence for GIV.1 NoVs detected in this investigation was lower than those reported for GI and GII NoVs in previous studies. In an earlier study in Italy, the seropositivity was 51.0% for GI.2 NoVs and 91.2% for GII.4 NoVs (*35*). In Japan, the seroprevalence rates were 82.0% for GI.1 NoVs and 88.0% for GII.3 NoVs (*36*), and in South Korea (*37*), the seroprevalence rates for GI.4, GII.3, and GII.4 NoVs were 84.1%, 76.3%, and 94.5%, respectively. However, consistent with the age-related patterns reported for GI and GII NoVs, we observed a positive association between the seropositivity for GIV.1 NoVs and age: the highest rates of antibodies were in children 5–10 years of age and in adults >50 years. GIV.1 NoVs have been documented in Italy, including in samples from children hospitalized with symptoms of acute gastroenteritis and in wastewater samples (*10*,*12*,*14*).

An age-related seroprevalence pattern was also observed for GIV.2 NoVs, although this profile differed from the seroprevalence profile of GIV.1 NoVs. The highest positive rate for GIV.2 NoV was detected in persons 15–35 years of age, although this pattern was not supported statistically. The overall prevalence rate for GIV.2 NoVs (4.8%) was markedly lower than the prevalence rates reported for other NoVs in humans (*27*,*31*,*37*), correlating with the lower prevalence of GIV viruses when compared with GII and GI viruses. Mesquita et al. (*34*) found IgG antibodies against GVI.2 viruses in 22.3% of small animal veterinarian practitioners and in 5.8% of the age-matched population controls, suggesting that veterinarians are more exposed to these viruses.

In conclusion, age-stratified serologic investigation revealed that GIV.1 NoVs are common in humans, although the prevalence of these viruses is somewhat low as documented in direct epidemiologic investigations. Also, antibodies specific for animal (GIV.2) NoVs were identified in human serum samples. This was not unexpected, because social interactions exist between humans and pets in all human populations and societies, and companion animals may transmit several zoonotic diseases to humans. Recent studies have also revealed that dogs can be infected, in turn, with human NoVs of different genotypes (*19*). Altogether, these findings seem to indicate that the evolution of human and animal NoVs is tightly intermingled.
